# Exposure assessment in early life: it is about time for multi-omics approaches

**DOI:** 10.1186/s12916-021-02088-0

**Published:** 2021-08-27

**Authors:** Jantje Goerdten, Anna Floegel

**Affiliations:** Unit Molecular Epidemiology, Department of Epidemiological Methods and Etiological Research, Leibniz Institute for Prevention Research and Epidemiology – BIPS, Achterstraße 30, 28359 Bremen, Germany

**Keywords:** Omics, Reliability, Variability, Exposure assessment, Children, DNA methylation, Gene expression, miRNA, Proteins, Metabolites

## Background

Multi-omics data, such as epigenomics, transcriptomics, proteomics, and metabolomics, are increasingly used to detect molecular responses to environmental exposures [1]. The integration of several omics layers can inform changes occurring in the structure, function, and dynamics of the body on a cellular level [2]. The correct identification of the effects of environmental exposures on the body is crucial in understanding disease development and progression [3]. Early life, i.e., pregnancy and childhood, is regarded as the most crucial periods of the developmental stages [4]. Environmental exposures in early life can have far reaching influences on the wellbeing and health of the individual [4]. Hence, the identification of these exposures and effects on the organism becomes critical.

### Variability of omics biomarkers in epidemiological studies

Omics biomarkers can pose objective measures of exposure, as they might be able to depict “true” exposure in individuals, i.e., the average exposure over a month or year [5]. Exposure biomarkers measure the extrinsic variables individuals are exposed to, for example, diet, tobacco smoke, pesticides, and air pollution. However, the technical and biological variability of omics profiles needs to be assessed. Epidemiological studies predominantly rely on single measurements [6]; hence, the biological variability of omics profiles should be known to interpret changes and classify individuals correctly. High variability can lead to biased results, namely misclassification bias which leads to incorrect effect estimates [7]. Variability can be influenced by the circadian rhythm, season, or individual characteristics and can be categorized into within-individual variability and between-individual variability [5]. Thereby, between-individual variability is desired to be higher than within-individual variability, so the investigated changes are due to differences between the subjects. Another important source of variability that has to be considered in omics studies is the technical variability that is derived from the laboratory methods and procedures [5]. This becomes a crucial issue when measuring numerous compounds with omics technologies in a large set of samples as in epidemiological studies. Technical variability in omics data includes random measurement errors that reduce statistical power [5], but also systematic measurement errors, such as batch effects, that lead to biased results. Technical variability needs to be addressed, e.g., by running quality controls, standardized procedures, normalization of the data, replication of the analysis, statistical adjustment, and proper randomization of the samples according to the study design [8].

### Studying variability of multi-omics layers in early life

In the present study of Gallego-Paüls et al. [9], the variability of six omics layers, namely blood DNA methylation, gene expression, miRNA, proteins, and serum and urine metabolites, from 156 children are evaluated. These children from six European cohort studies participate in the Human Early-Life Exposome (HELIX) project and have been sampled at two time points around 6 months apart. This study comprehensively assesses the within-individual and between-individual variability of each omics layer, evaluates the interrelationships between the variability in the omics layers, and analyzes the influence of several factors on the variability. The authors report large heterogeneity between the omics layers and their variability. Age, body-mass-index (BMI), and hour of collection are the characteristics with the highest influence on the variability, while variability due to cohort membership is small. DNA methylation and serum metabolites are the most stable features, with high between-individual variability and lower within-individual variability. Proteins and urinary metabolites present somewhat more variability; however, they show great heterogeneity between the features. Lastly, gene expression presents the highest variability and is therefore the least stable omics layer.

The HELIX project set out to establish the early life exposome which includes the complete set of exposures individuals are exposed to during pregnancy and childhood [3]. To correctly measure all exposures during early life, the uncertainty in exposure estimates needs to be assessed. Therefore, each omics layer should be reliable, i.e., present small variability over time in an individual. These results show the various variability profiles of the omics layers and their features in European children. The discovered influences on variability advance the understanding and interpretation of individual omics changes in children. Nevertheless, these results are based on a small sample of children and need to be replicated in larger and broader populations of children. The great strengths of this study are the inclusion of healthy children from different European countries and the evaluation of several omics layers. On the other hand, only a targeted analysis was performed for proteomics and metabolomics, and the use of untargeted analysis would result in a broader picture of all features belonging in the different layers. Furthermore, no technical replicate samples were analyzed in the laboratory; hence, the technical variability was only to some extent controlled for. Last but not the least, dietary intake on the day before sample collection was not controlled for; however, it may particularly impact urinary metabolites and may be one reason for the lower reliability observed here.

## Conclusion

The study by Gallego-Paüls et al. demonstrates that single measurements of DNA methylation and serum metabolites can be used to depict exposure in children over 6 months. In contrast, proteins, urinary metabolites, and gene expression are rather used to depict a short-term exposure in children. However, for proteins and urinary metabolites, it is also highly dependent on the individual compounds as some of them are more reliable than others. The variability of the omics profiles can be reduced by adjusting for age and BMI and by following standard protocols for sample collection. Despite some limitations, the present study reports on an important topic, i.e., the omics-based exposure assessment in children, which has been understudied. It further paves the road for future large epidemiological studies that plan to include multiple omics layers.

## Data Availability

Not applicable.
